# Evaluation of lysyl oxidase-like 1 gene polymorphisms in pseudoexfoliation syndrome in a Korean population

**Published:** 2013-02-22

**Authors:** Do Young Park, Hong-Hee Won, Hyun-kyung Cho, Changwon Kee

**Affiliations:** 1 Department of Ophthalmology, Samsung Medical Center, Sungkyunkwan University School of Medicine, Seoul, Korea; 2 Samsung Biomedical Research Institute, Samsung Medical Center, Sungkyunkwan University School of Medicine, Seoul, Korea

## Abstract

**Purpose:**

The purpose of this study was to evaluate association profiles of lysyl oxidase-like 1 (*LOXL1*) gene polymorphisms with pseudoexfoliation syndrome (XFS) in a Korean population.

**Methods:**

A total of 110 Korean patients with XFS and 127 control subjects were included in this study. Genotypes of three single nucleotide polymorphisms (SNPs) of *LOXL1* (rs1048661, rs3825942, and rs2165241) were analyzed with direct sequencing, and a case-control association study was performed. Genotype frequencies of each SNP were compared according to the XFS phenotypes.

**Results:**

All three SNPs were significantly associated with XFS. The T allele at rs1048661 (odds ratio [OR]=14.29, 95% confidence interval [CI]=6.25–33.3) and the C allele at rs2165241 (OR=7.14, 95% CI=1.59–33.3) were risk alleles in Korean subjects, which was consistent with findings in other Asian populations. However, our findings were opposite to results from Caucasian populations in which the risk alleles at rs1048661 and rs2165241 were G and T, respectively. At the rs3825942, the G allele (OR=12.50, 95% CI=2.94–50.0) was a risk allele for XFS, which was similar to results from most other ethnic groups except black South Africans in whom the A allele increased the risk. In the haplotype analysis, the T-G-C haplotype composed of all three risk alleles was significantly overrepresented in XFS and conferred an 11.36 fold (95% CI=5.97–23.49) increased likelihood of XFS. There was no significant association between the genotype frequencies of the three SNPs and the XFS phenotypes.

**Conclusions:**

Three SNPs of *LOXL1* (rs1048661, rs3825942, and 2,165,241) are highly associated with XFS in a Korean population. The risk alleles of these SNPs were similar to those of other Asian populations, such as Japanese or Chinese, but differed from non-Asian populations, suggesting that still unidentified genetic or environmental factors may contribute to disease expression.

## Introduction

Pseudoexfoliation syndrome (XFS) is an age-related systemic disorder of the extracellular matrix characterized by production and progressive accumulation of fibrillar material in many ocular tissues [1]. XFS is the most common identifiable cause of open angle glaucoma worldwide [2]. The conversion rate of XFS to pseudoexfoliative glaucoma (XFG) was reported at 44% over 15 years [3]. XFG is characterized by rapid progression of glaucomatous optic nerve damage, high resistance to medical therapy, and a worse prognosis than primary open angle glaucoma [1].

A recent genome-wide association study in an Icelandic population demonstrated that three single nucleotide polymorphisms (SNPs) in the lysyl oxidase-like 1 (*LOXL1*) gene on chromosome 15q24.1 have a strong association with XFS, two nonsynonymous coding SNPs (rs1048661[R141L] and rs3825942[G153D]) in exon 1 of *LOXL1* and one intronic SNP (rs2165241), and a population attributable risk of more than 99% [4]. Since then, the association of *LOXL1* SNPs with XFS/XFG has been reported in various ethnic groups in various regions, including North America [5-9], Australia [10], Europe [11,12], South Africa [13,14], and Asia [15-22]. However, the association studies’ results vary among races. For example, the risk allele of rs1048661 has been reported to be different between Asian and Caucasian populations. These disparities among different populations were also observed even in Asian populations. In Indian [21] and Chinese [18] populations, the G allele of SNP rs1048661 was not associated with XFS/XFG. Other reports from Japan [16,17,20,22] and China [15] demonstrated that the opposite T allele of SNP rs1048661 increased the risk for XFS. Thus, the present study was designed to confirm the association of three SNPs in the *LOXL1* gene with XFS in a Korean population and to compare results to previous studies of other ethnic groups.

## Methods

### Study subjects

A total of 110 unrelated patients with XFS and 127 control subjects who were healthy and aged more than 21 years old were recruited from the Glaucoma Clinic at the Samsung Medical Center, Sungkyunkwan University School of Medicine. 52 male and 58 female patients with XFS were recruited from May 2011 to April 2012. Among 127 control subjects recruited for the same time period 56 were male and 71 were female recruited from the Glaucoma Clinic at the Samsung Medical Center. Patients with XFS were identified by the presence of pseudoexfoliative material on the anterior lens capsule after maximal pupil dilatation. XFG was diagnosed if patients had typical features of XFS and all of the following: an initial intraocular pressure of at least 22 mmHg, glaucomatous optic disc changes, visual field defects consistent with optic nerve damage, and no evidence of other conditions causing secondary glaucoma. All subjects underwent complete ophthalmic examinations including slit-lamp biomicroscopy examination, gonioscopy, and funduscopy. The control group included 127 unrelated healthy Koreans who did not show any clinical evidence of XFS. All patients with XFS and control subjects were ethnically Korean and age- and sex-matched. A diagnosis of glaucoma was ruled out through examination of intraocular pressure and the optic disc in all members of the control group. The study was conducted in compliance with the tenets of the Declaration of Helsinki for the use of human subjects in biomedical research, and institutional review board approval was obtained. Written informed consent was obtained from all study participants.

### Lysyl oxidase-like 1 gene analysis

Peripheral blood (3~5 ml each) was collected in tubes containing EDTA from 110 patients with XFS and 127 control subjects with informed consent. The blood samples were stored at -80°C immediately after collection until use. Genomic DNA (gDNA) was extracted from the frozen blood using Wizard gDNA purification kit (promega, Medison, WI) according to the manufacturer's protocol. Three single nucleotide polymorphisms (rs1048661, rs3825942, and rs2165241) of *LOXL1* were investigated in the patients with XFS and the control group. Genotyping of SNPs was performed using the 5′ exonuclease assay (TaqMan; Applied Biosystem, Inc. [ABI], Foster City, CA). The fluorescence signal of the probe was detected with real-time PCR (TaqMan assay for real PCR [RT–PCR], 7000 Real-Time PCR Systems; ABI).

### Statistical analysis

We removed samples with a call rate of <50%, and SNPs with a call rate of <80%, a minor allele frequency <0.01, and Hardy–Weinberg equilibrium (HWE) p value <0.001. HWE was checked using Haploview 4.1 [23]. The association between each SNP and XFS was tested with the Cochran-Armitage trend test in the additive model and by Fisher’s exact test in the dominant (minor homozygous and heterozygous versus major heterozygous) or recessive model (minor homozygous versus heterozygous and major heterozygous) using PLINK 1.07 [24]. Allelic odds ratios were estimated. In addition, genotype frequencies of each SNP were compared between subgroups in the patients with XFS according to their related phenotypes (bilaterality or development of XFG) using Fisher’s exact test. Multiple logistic regression analysis with three SNPs was performed using PLINK 1.07. For haplotype analyses, haplotypes of samples were constructed using PHASE v2.1 [25]. The association of each haplotype (one haplotype versus other haplotypes) was tested with Fisher’s exact test using the R 2.15.1 software.

## Results

The present study included 110 patients with XFS (52 male, 58 female) and 127 control subjects (56 male, 71 female). Of the 110 patients with XFS, 45 had XFG. Bilateral involvement of XFS was found in 20 patients among those with XFS. The mean±standard deviation (SD) age of the XFS and control groups was 71.6±9.8 and 70.2±7.5 years, respectively. There was no significant difference in mean age between the XFS and control groups (p>0.05).

Before conducting the association analyses, we checked the quality of the 237 samples and three SNPs of *LOXL1*. All samples showed a call rate of >50%, and all three SNPs showed a call rate of >90%. (The call rate for rs1048661, rs3825942, and rs2165241 was 100%, 100%, and 91%, respectively.) No SNPs were significantly deviated from HWE in the case and control samples. The minor allele frequencies of the SNPs were greater than 1%. Trend analysis of allele frequency revealed that the number of T allele of rs1048661 was significantly associated with an increased risk for XFS (odds ratio [OR]=14.29, p=2.13×10^−12^; Table 1). The G allele of rs3825942 and the C allele of rs2165241 were also significantly associated with XFS (OR=12.50, p=9.12×10^−6^ for the G allele of rs3825942, OR=7.14, p=2.59×10^−3^ for the C allele of rs2165241). The association remained significant after the Bonferroni correction. Linkage disequilibrium (LD) values (*r^2^*) of the two SNPs (rs3825942 and rs2165241) with the top SNP rs1048661 were 0.113 and 0.022, respectively. Each minor allele of the SNPs was less frequent (about tenfold) in the patients with XFS compared to the control subjects.

**Table 1:** Allele Frequencies of SNPs of *LOXL1* and Association Results

**SNPs**	**Minor allele**	**Major allele**	**MAF**		***P***	**OR (95% CI)**
			**Control (n=127)**	**Case (n=110)**		
rs1048661 (R141L)	G	T	29.5	2.7	2.13×10^−12^	14.29 (6.25–33.33)
rs3825942 (G153D)	A	G	10.2	0.9	9.12×10^−6^	12.50 (2.94–50.00)
rs2165241	T	C	6.5	1.0	2.59×10^−3^	7.14 (1.59–33.33)

SNPs, single nucleotide polymorphisms; MAF, minor allele frequency; OR, allelic odds ratio; CI, confidence interval. *P*, by Cochran-Armitage trend test

We also tested the association in the dominant and recessive models. Significance of the association of three SNPs increased in the dominant model (Table 2) compared to the additive model but decreased in the recessive model (data not shown). This suggests that having at least one minor allele might be protective against XFS.

**Table 2:** Genotype frequencies of the SNPs of *LOXL1* in a Dominant Association Model

**SNPs**	**Genotype frequency (Minor homozygosity +heterozygosity*/*major homozygosity)**		***P***	**OR (95% CI)**
	**Control (n=127)**	**Case (n=110)**		
rs1048661 (GG+TG */* TT)	62 / 65	5 / 105	1.42×10^−15^	19.80 (7.49–66.40)
rs3825942 (AA+GA */* GG)	26 / 101	2 / 108	3.30×10^−6^	13.79 (3.31–123.02)
rs2165241 (TT+CT */* CC)	15 / 100	2 / 99	2.22×10^−3^	7.37 (1.65–68.22)

SNPs, single nucleotide polymorphisms; OR, odds ratio; CI, confidence interval. *P*, by Fisher’s exact test.

We also performed multiple logistic regression analyses with three SNPs. The most significant SNPs rs1048661 (p=2.86×10^−6^) and rs2165241 (p=0.0277) were significant, but rs3825942 was not significant (p=0.132), which indicates that a combination of multiple SNPs in this region may cause an additional association effect.

Haplotype association analysis identified three common haplotypes (T-G-C, G-G-C, and G-A-C) with a frequency of >5%, defined by three SNPs (rs1048661-rs3825942-rs2165241). The estimated haplotype frequencies are presented in Table 3. The haplotypes were significantly associated with XFS either as a risk haplotype or a protective haplotype. The T-G-C haplotype, composed of the risk alleles of the three SNPs, conferred an 11.36-fold increase in the risk of XFS. Only the T-G-C haplotype was more frequently involved in patients with XFS compared to controls while other haplotypes were less frequent in the patients with XFS than in the controls.

**Table 3:** Haplotype Analysis

**Haplotype**	**Haplotype Frequency**		***P***	**OR (95% CI)**
	**Control (n=127)**	**Case (n=110)**		
T-G-C	161 (61.0%)	214 (94.7%)	2.47×10^−20^	11.36 (5.97–23.49)
G-G-C	54 (20.5%)	2 (0.9%)	1.57×10^−13^	0.03 (0.00–0.14)
G-A-C	26 (9.8%)	8 (3.5%)	6.95×10^−3^	0.34 (0.13–0.79)

OR, odds ratio; CI, confidence interval. *P*, by Fisher’s exact test.

Subgroup analysis revealed that three SNPs of *LOXL1* were not associated with other relevant phenotypes of XFS, including bilateral involvement of XFS or the development of glaucoma (p>0.05).

## Discussion

Consistent with previous studies of various ethnicities, we also found a strong genetic association between polymorphisms of *LOXL1* and XFS in a Korean population. The *LOXL1* gene is a member of the lysyl oxidase family of extracellular enzymes necessary for specific oxidative deamination of lysine residues and cross-linking of elastin polymers. The N-terminal of the *LOXL1* protein contains the catalytic domain, which has multiple functions including enzyme activation, substrate recognition, and binding [26]. In previous studies [27], mice deficient in the *LOXL1* gene showed reduced elastin content in multiple tissues leading to pelvic organ prolapse, emphysematous changes, and vascular abnormalities. These findings imply that the *LOXL1* protein has an essential role in the homeostasis of elastic fiber. Therefore, it is plausible that altered *LOXL1* function could increase the susceptibility for XFS.

In a genome-wide association study, Thorleifsson et al. [4] identified a strong association between three *LOXL1* SNPs and XFS in Swedish and Icelandic populations. This association was confirmed in several different populations including North Americans [5-9], Australians [10], Europeans [11,12], South Africans [13,14], and Asians [15-22]. However, the frequency and type of allele conferring increased risk for XFS for the three *LOXL1* SNPs varied among different ethnic groups. For rs1048661, the G allele was reported as a risk allele for XFS in most Caucasians including Nordic, American, German, Italian, Australian, and Finnish populations [4,8,10,12,28], while the opposite T allele was reported to increase the risk of XFS in Asian populations such as Japanese and Chinese [15,16,19]. For rs3825942, the G allele was significantly associated with XFS in most populations including Caucasians and Asians [4,7,8,10,12,15,18,19,21,28], but recent reports on black South Africans revealed that the opposite A allele was associated with XFS [13,14]. For rs2165241, the T allele was reported as a risk allele in Caucasians [4-7,12], while the C allele was reported to increase the risk for XFS in Japanese and Chinese populations [15,20,22].

In the present study, the T allele of rs1048661, the G allele of rs3825942, and the C allele of rs2165241 were significantly associated with increased risk of XFS in a Korean population. A recent report by Sagong et al. [29] of Korean patients corroborates the findings of this study. One of the differences between this study and the previous study is that a patient with any type of glaucoma was excluded from the control group in our study. Some patients with glaucoma, such as primary open angle glaucoma, chronic angle closure glaucoma, or secondary glaucoma, were included in the control group in Sagong et al.’s study, and exclusions in this study resulted in a more homogenous control group. In the present study, the minor allele frequency (MAF) of the most significant SNP rs1048661 in the control group (0.295) was much lower than those from other Asian populations including Japanese and Chinese [15-17,20], ranging from 0.460 to 0.497 despite the same minor allele of G, which may have been the result of different ethnic backgrounds.

Based on results of *LOXL1* polymorphisms in Korean populations, we confirmed that the allele frequencies of rs1048661 and rs2165241 in a Korean population show similar patterns with other Asian populations, including Japanese and Chinese, but opposite results of other ethnic groups, including Caucasians. Similarly, we found that the allele frequency of rs3825942 revealed a similar pattern with most populations including Caucasians and Asians, while analysis showed different patterns in black South Africans. These discrepancies in genetic findings among different ethnic groups suggest that missense changes in these SNPs of *LOXL1* are not directly responsible for the development of XFS; rather, other unidentified genetic or environmental factors independent of *LOXL1* may affect gene expression or protein function, which needs further investigation. Fan et al. [30] recently demonstrated that *LOXL1* promoter haplotypes, which may affect *LOXL1* expression and enzyme activity, were significantly associated with XFS/XFG in a U.S. Caucasian population. Comparing promoter SNPs in other ethnic groups, such as a Korean population, may help to explain these discrepancies seen in *LOXL1* SNPs and the development of XFS in different ethnic groups.

In the haplotype-based association analysis, only the T-G-C haplotype showed an increased risk for XFS in the Korean population, which was consistent with the results from other studies analyzing Asian populations [15,16,20]. The G-G-C haplotype was significantly associated with XFS as a protective haplotype in the Korean population, whereas the G-G haplotype defined by rs1048661 and rs3825942 was known to be a risk haplotype in Caucasian populations [6,10,12]. The G-A haplotype defined by rs1048661 and rs3825942 was the only haplotype found to be associated with an increased XFS risk for XFS in black South African populations [13,14] but revealed a protective effect in our study. According to a report by Kim et al. [31], haplotype variants of *LOXL1* defined by rs1048661 and rs3825942 did not affect the amine oxidase activity of *LOXL1*. This finding is consistent with genetic findings of different risk haplotypes among different populations.

In this study, we divided the XFS group by phenotype (XFS without glaucoma and XFG, unilateral and bilateral XFS) and compared the allelic associations of the three *LOXL1* SNPs to the different XFS subgroups. In accordance with the results of previous studies [4,20,29], there was no significant difference in the genotypic frequencies of the three SNPs when different XFS phenotypes were considered. These results suggest that the *LOXL1* polymorphism is not associated with the progression to different XFS phenotypes; instead, other unidentified genetic or environmental factors may influence the expression of the different XFS manifestations. One example of genetic factors is *CDKN2BAS*, which recently was reported to be associated with XFG [32]. *CDKN2BAS* is related to transforming growth factor beta signaling, which has a role in glaucomatous optic nerve disease and retinal ganglion cell death [32].

In summary, the results of our study confirmed a significant association of the three SNPs with XFS in a Korean population. The risk alleles and haplotypes of the three SNPs were consistent with Asian populations, including Japanese and Chinese, while previously described differences with Caucasians were observed. These SNPs were not significantly associated with the phenotypes of XFS, including bilateral XFS or the development of glaucoma. The pathophysiologic role of *LOXL1* for XFS has not been completely described, and further investigations are needed to determine additional genetic or environmental XFS risk factors.
